# High-Quality Genome Assembly and Comprehensive Transcriptome of the Painted Lady Butterfly *Vanessa cardui*

**DOI:** 10.1093/gbe/evab145

**Published:** 2021-06-28

**Authors:** Linlin Zhang, Rachel A Steward, Christopher W Wheat, Robert D Reed

**Affiliations:** 1 CAS and Shandong Province Key Laboratory of Experimental Marine Biology & Center of Deep Sea Research, Center for Ocean Mega-Science, Institute of Oceanology, Chinese Academy of Sciences, Qingdao, China; 2 Laboratory for Marine Biology and Biotechnology, Qingdao National Laboratory for Marine Science and Technology, Qingdao, China; 3 College of Earth Science, University of Chinese Academy of Sciences, Beijing, China; 4 Department of Zoology, Stockholm University, Stockholm, Sweden; 5 Department of Ecology and Evolutionary Biology, Cornell University, Ithaca, New York, USA

**Keywords:** PacBio sequencing, de novo genome assembly, RNA-seq, butterfly wing, color patterning

## Abstract

The painted lady butterfly, *Vanessa cardui*, has the longest migration routes, the widest hostplant diversity, and one of the most complex wing patterns of any insect. Due to minimal culturing requirements, easily characterized wing pattern elements, and technical feasibility of CRISPR/Cas9 genome editing, *V. cardui* is emerging as a functional genomics model for diverse research programs. Here, we report a high-quality, annotated genome assembly of the *V. cardui* genome, generated using 84× coverage of PacBio long-read data, which we assembled into 205 contigs with a total length of 425.4 Mb (N50 = 10.3 Mb). The genome was very complete (single-copy complete Benchmarking Universal Single-Copy Orthologs [BUSCO] 97%), with contigs assembled into presumptive chromosomes using synteny analyses. Our annotation used embryonic, larval, and pupal transcriptomes, and 20 transcriptomes across five different wing developmental stages. Gene annotations showed a high level of accuracy and completeness, with 14,437 predicted protein-coding genes. This annotated genome assembly constitutes an important resource for diverse functional genomic studies ranging from the developmental genetic basis of butterfly color pattern, to coevolution with diverse hostplants.

Significance

*Vanessa cardui* is a widely distributed butterfly species and has emerged as an excellent model for studying color pattern formation, migration, and coevolution. Here, we present a high-quality, annotated reference genome of *V. cardui*. This new genome assembly will serve as an important tool for genome-scale functional studies in *V. cardui* and a resource for advancing research in evolution, development, and ecology.

## Introduction

The painted lady butterfly, *Vanessa cardui* (Linnaeus 1758), is one of the most widely distributed butterfly species (Shields 1992). It occurs from sea level to about 5,200 m in elevation on every continent except Antarctica and South America (Shields 1992; Varshney and Smetacek 2015). *Vanessacardui* is a long-range, seasonal migratory butterfly that undertakes an annual multigenerational migration across most of Europe in spring and summer, and north Africa in autumn and winter (Stefanescu et al. 2007; Stefanescu, et al. 2013; Stefanescu et al. 2016, 2017; Pfeiler and Markow 2017).*V. cardui* is also actively studied for its hostplant interactions (de la Paz Celorio-Mancera et al. 2016; Gamberale-Stille et al. 2019), visual biology (Briscoe et al. 2003; Briscoe and White 2005; Perry et al. 2016), and thermoregulation (Tsai et al. 2020).

*Vanessa* *cardui* has also emerged as an excellent model for studying color pattern formation (Reed and Nagy 2005; Hiyama et al. 2012; Dinwiddie et al. 2014; Connahs et al. 2016). Melanins and ommochromes, the pigment types characteristic of the major butterfly family Nymphalidae, are diverse and abundant in this species, and *V. cardui* wings display all of the major pattern elements of the Nymphalid Ground Plan (Nijhout 1991). *Vanessacardui* is also highly accessible for both classroom projects (Martin et al. 2020) and lab studies because it is readily available from commercial vendors and can be reared in large numbers on an artificial diet. Recently, CRISPR/Cas9 genome editing tools have become established in *V. cardui*, which allows for straightforward experimental validation of gene function. CRISPR/Cas9 knockout studies carried out in *V. cardui* have identified color patterning (*optix*, *WntA*, *distal-less*, *spalt*) (Zhang and Reed 2016; Mazo-Vargas et al. 2017; Zhang et al. 2017b) and pigmentation genes (*pale*, *Ddc*, *yellow*, *yellow-d*, *yellow*, *ebony*, *black*) (Perry et al. 2016; Zhang et al. 2017a). In sum, *V. cardui* is attracting increasing attention in the field of developmental genetics, ecology, and evolutionary biology as a model for connecting genotypes to diverse phenotypes and is thus a powerful addition to comparative studies.

Lepidoptera are a diverse order of insects with complex morphological and behavioral traits, and work on this group will benefit from more and better genomic resources. *Vanessacardui* belongs to the Nymphalidae, which is the largest family of butterflies. There are currently seven annotated nymphalid genomes accessible on the public genome browser Lepbase (Challi et al. 2016) ( http://lepbase.org/, May 18, 2021): *Heliconius erato* (Lewis et al. 2016; Van Belleghem et al. 2017), *Heliconius melpomene* (Dasmahapatra et al. 2012), *Bicyclus anynana* (Nowell et al. 2017), *Melitaea cinxia* (Blande et al. 2020), *Calycopis cecrops* (Cong, Shen, Borek, et al. 2016), *Junonia coenia* (van der Burg et al. 2019), and *Danaus plexippus* (Zhan et al. 2011). This paper adds to this list by reporting a high-quality *V. cardui* genome assembly, generated using PacBio long-read sequencing technology. The final assembly was 425.4 Mb in length, with a contig N50 of 10.3 Mb. We further performed deep transcriptomic sequencing and analyzed 29 RNA-seq data sets across multiple tissues and developmental stages. Using the genome assembly and transcriptomic resources, we annotated protein-coding genes and repeat sequences. The resulting genome assembly, annotation, and wing development expression profiles will provide a valuable resource for future studies of the painted lady butterfly and for butterfly and insect biology in general.

## Results and Discussion

### High-Quality Genome Assembly

**Fig. 1:**
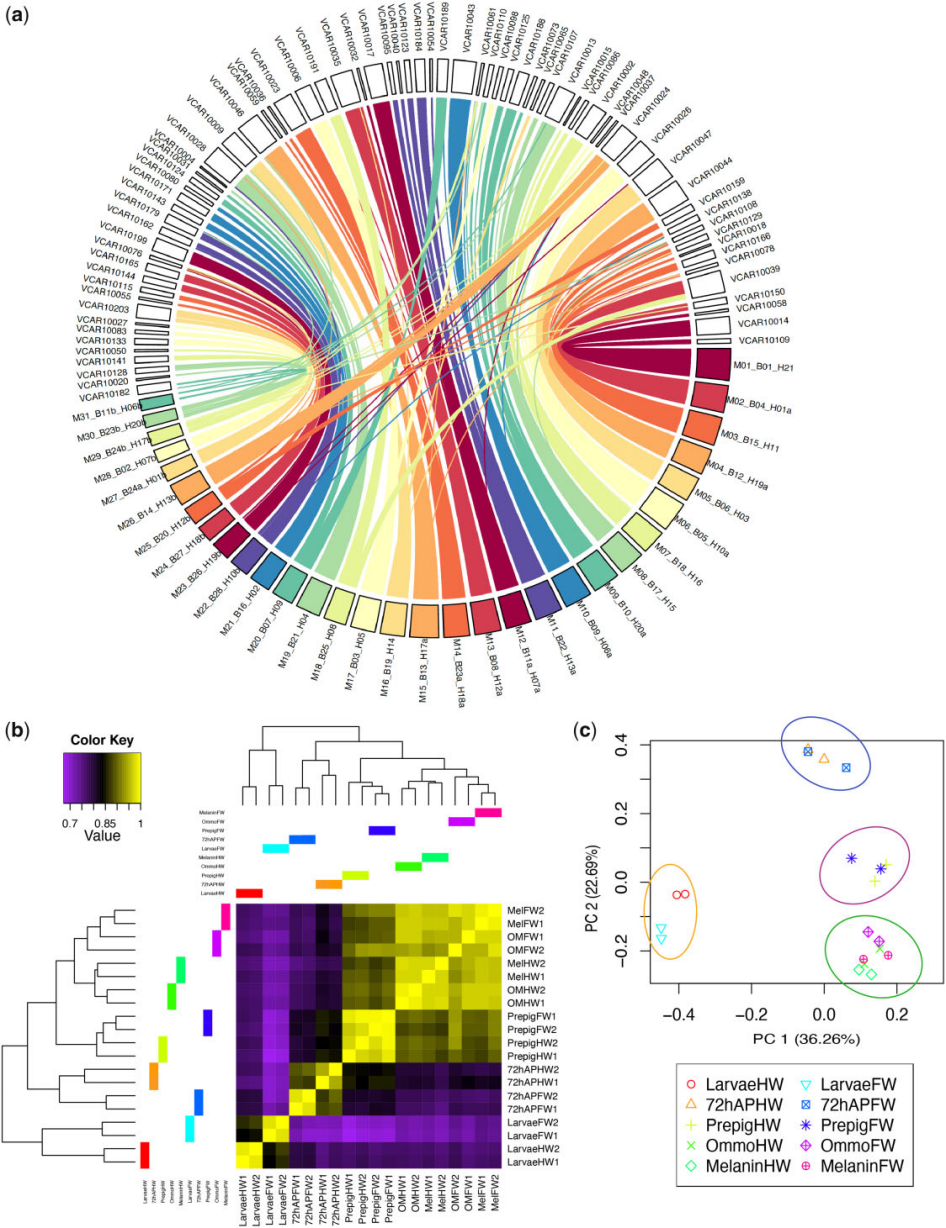
*Vanessa cardui* genome synteny and transcript clustering. (*a*) Synteny of corresponding chromosomes between *V. cardui* and *Melitaea cinxia*. Homologous regions of the genome assemblies are connected by colored lines that represent syntenic regions identified by MUMmer. (*b*) Heatmap of gene expression clustering by replicate (1, 2), tissue type (FW: forewing, HW: hindwing), and developmental stage (last instar larvae, 72 h after pupation, prepigmentation, ommochrome stage, melanin stage). (*c*) Principal component analysis of gene expression.

A total of 36.53 Gb of PacBio long reads (coverage of 84×) were generated from 55 SMART cells. The total length of the genome assembly of *V. cardui* was 425.41 Mb with a contig N50 of 10.30 Mb (table 1). We further generated *V. cardui* pseudochromosomes using a high-quality chromosomal assembly from *M. cinxia* (v2) (Blande et al. 2020), which is the closest related nymphalid with a high-quality assembly. The final pseudochromosome assembly contained 143 contigs with the N50 of 15.35 Mb ( fig. 1*a*). The completeness of our assembly was assessed by Benchmarking Universal Single-Copy Orthologs (BUSCO). Using Lepidoptera-specific single-copy orthologs (lepidoptera_odb10), 96.9% and 0.7% of 5,286 BUSCOs were complete and partially assembled, respectively, with only 0.3% duplicated. Overall, all evidence suggests that the *V. cardui* assembly is a high-quality genome assembly that can be used for further downstream analyses.

**Table 1:** *Vanessa cardui* Genome Assembly and Annotation Summary

**Genome assembly statistics**	
Total length (bp)	425,413,715
Contig N50 length (bp)	10,297,021
Contig N90 length (bp)	1,988,721
Longest contig length (bp)	15,944,461
Number of contigs	205
Number of contigs larger than N50	16
Number of contigs larger than N90	54
**Genome characteristics**	
GC content	33.37%
Number of protein-coding genes	14,437
Average transcript length (bp)	7,947.27
Average CDS length (bp)	1,285.78
Average exon length	208.90
Average exons per gene	6.26
**Repetitive sequences (% of genome)**	
DNA (bp)	26,747,187 (6.29%)
LINE (bp)	44,319,571 (10.42%)
SINE (bp)	36,688,707 (8.62%)
LTR (bp)	7,782,116 (1.83%)
Simple repeat (bp)	7,080,895 (1.66%)
Unknown (bp)	23,180,775 (5.45%)
Total (bp)	142,884,949 (33.59%)
**Gene annotations (% of all genes)**	
SwissProt	13,751 (95.25%)
KEGG	8,153 (56.47%)
GO	9,563 (66.24%)
PFAM	12,000 (83.12%)
InterProScan	10,533 (72.96%)
Total	14,097 (97.64%)

### Repeat and Gene Annotation

We identified a total length of 144,928,423 bp repeat sequences, accounting for 34.07% of *V. cardui* genome (table 1). The most abundant of the transposable and repetitive element type was long interspersed nuclear elements (LINE), representing 44.32 M (10.42%) of the genome. A gene set of 14,437 protein-coding genes was generated with a mean of 6.16 exons per gene (table 1). A total of 14,097 protein-coding genes (97.64%) were successfully annotated for at least one function term by searching against functional databases (SwissProt, gene ontology [GO], Kyoto Encyclopedia of Genes and Genomes [KEGG], PFAM, and InterProScan) (table 1). In order to test the quality of gene annotation, we compared ortholog hit ratios between our final *V. cardui* annotation with that from *Bombyx mori* and *D. plexippus*. More than 90% of the 14,439 *B. mori* query proteins had orthologous alignments against annotations from both *V. cardui* and *D. plexippus*, suggesting both annotations are very complete (supplementary fig. S1 and S2, Supplementary Material online).

### Phylogenetic Analysis

To confirm the phylogenetic position of *V. cardui* and estimate divergence times using whole-genome data, we analyzed the orthologous gene relationships between *V. cardui* and 12 other lepidopterans. The phylogenetic analysis suggests that butterflies originated from moths around 85–177 Myr and Nymphalidae started diversifying around 85–131 Myr. These results broadly agree with a previous study’s confidence intervals (Espeland et al. 2018). Of the species examined, *V. cardui* is most closely related to *M. cinxia*, and the two species diverged from the *H. melpomene* lineage ∼73–84 Myr (supplementary fig. S3, Supplementary Material online).

### Gene Expression Analysis

To explore the molecular basis of the butterfly wing developmental process, we generated a comprehensive profile of gene expression across wing developmental stages from both forewings and hindwings (supplementary table S1, Supplementary Material online, and fig. 1*b*). The first principal component explained 36.36% of the variance in gene expression and showed strong separation at larval and pupal stages, highlighting the different development processes occurring at these wing developmental stages (fig. 1*c*). We further performed differential gene expression analysis by comparing consecutive developmental stages. Overall, we identified 2,305 genes significantly differentially expressed (false discovery rate [FDR] < 0.001) (supplementary fig. S4, Supplementary Material online) including 1,692 genes identified from forewing and 1,806 from hindwing transcriptomes (supplementary table S3, Supplementary Material online). The gene set provides a useful resource to further explore the molecular genetic underpinnings of butterfly wing pattern evolution.

## Materials and Methods

### Sample Collection and Sequencing

*Vanessa* *cardui* butterflies were purchased from Carolina Biological Supply. They were fed on a multispecies artificial diet (Southland) and maintained in a 16:8 h light/dark cycle at 28 °C. Total genomic DNA of a single female *V. cardui* was extracted from a prepigmentation stage pupa using a QIAGEN Genomic-tip kit. We applied PacBio single-molecule, real-time (SMRT) sequencing system for DNA library construction and sequencing.

*Vanessa* *cardui* whole-body and wing tissue samples were collected for RNA library construction and sequencing. *Vanessacardui* were first sampled at multiple developmental stages, including early embryonic development (<12 h postoviposit), late embryonic to early larval development (12–52 h postoviposit), and hatched larva (mixture with early, middle-, and late-stage larvae). *Vanessacardui* pupal tissues were also collected along the anterior–posterior body axis (head, thorax, and abdomen, respectively) from both early stage (i.e., 3 days after pupation) and late melanin-stage pupae (i.e., ∼6 days after pupation when black melanin pigments began to show up). Second, forewings from five different wing developmental stages of *V. cardui* were sampled (supplementary table S1, Supplementary Material online), including last instar larvae, 3 days after pupation, prepigmentation stage (∼5 days after pupation), ommochrome development (∼5.5 days after pupation when red–orange ommochrome pigments started to show up), and melanin development pupae. Hindwings across multiple wing developmental stages were previously sampled (Zhang et al. 2017a). Two biological replicates of each wing developmental stage were prepared. Total RNA was extracted from each sample with an Ambion Purelink RNA Mini Kit (Life Technologies). RNA libraries were constructed using the NEBNext Ultra RNA Library Prep kit for Illumina (New England Biolabs).

### Genome Assembly and Assessment

Whole-genome SMRT data of *V. cardui* was first passed through TANmask and REPmask modules from the Damasker suite. The initial error-corrected reads were then processed by the overlap portion of the FALCON pipeline (Chin et al. 2016) using a length cutoff of 5,000bp. After assembly, the genome was polished by Quiver using the original raw reads. HaploMerger2 (Huang et al. 2017) was run to produce an improved, deduplicated assembly. In addition, we aligned the *V. cardui* genome against *M. cinxia* genome reference for chromosome assembly. Using MUMmer alignment package (Marçais et al. 2018), we generated one-to-one alignments of best hits between these two genomes with an alignment identity of between 80% and 90%, for regions of at least 200 bp in length, for scaffolds of ≥1 Mb in length. A circle plot of the alignment was made using custom R scripts, with packages tidyverse v1.3.0 (Wickham et al. 2019), circlize v0.4.10 (Gu, et al. 2014) and RColorBrewer v1.1-2. We used BUSCO (Simão et al. 2015) to evaluate the genome completeness. We compared the assembled and structural annotation metrics of *V. cardui* with those of other butterfly species for further evaluation (supplementary table S2, Supplementary Material online).

### Annotation of Repetitive Elements

Genome sequences were analyzed with RepBase (v20181026) (Bao et al. 2015) to identify repeats using RepeatMasker (v4.0.6) (Bergman and Quesneville 2007) and RepeatProteinMask (-noLowSimple *P* value 0.0001). Tandem repeat finder (v4.09) (Benson 1999) was used to identify tandem repeats. In addition, RepeatModeler (v1.0.9) (Flynn et al. 2020) was employed to construct a de novo repeat library. This species-specific library was subsequently utilized to detect repeat sequences with RepeatMasker in the *V. cardui* genome.

### Gene Prediction, Functional Annotation, and Assessment

We employed three different approaches to predict protein-coding genes. First, homology-based annotation was performed by TBLASTN (Camacho et al. 2009) using protein sequences from six related species including *Heliconius erato* (Lewis et al. 2016), *H. melpomene* (Davey et al. 2016), *B. anynana* (Nowell et al. 2017), *D. plexippus* (Zhan et al. 2011), *Phoebis sennae* (Cong, Shen, Warren, et al. 2016), and *Papilio xuthus* (Li et al. 2015). GeneWise v2.4 (Birney et al. 2004) was then employed to align against the matching protein for the accurate spliced alignment and gene structure prediction. Second, transcriptome-based annotation was applied by both de novo and reference-guided approaches. With the 34.24 Gb of RNA sequence data generated from the 29 samples described above (supplementary table S1, Supplementary Material online), de novo transcript assembly was performed by Trinity pipeline v2.4.0 (Grabherr et al. 2011). For the reference-guided approach, RNA reads were mapped onto the *V. cardui* genome assembly using Tophat v2.1.1 (Trapnell et al. 2009). Subsequently, Cufflinks v2.2.1 (Trapnell et al. 2010) and cuffmerge were employed to assemble the mapped reads and predict the structure of all transcribed reads with the default parameters. The predicted gene sets generated from de novo and reference-guided approaches were then integrated to produce nonredundant empirical transcript evidence by Program to Assemble Spliced Alignment v2.0.2 (Haas et al. 2003). Third, ab intio gene prediction were carried out on the repeat-masked *V. cardui* genome assembly using Scalable Nucleotide Alignment Program v 2006-07-28 (Korf 2004) and Augustus v3.2.3 (Stanke and Waack 2003). Gene models from homology-based and transcriptome-based annotation were trained for gene prediction. Finally, MAKER v 2.31.8 (Campbell, et al. 2014) was used to combine homology, transcriptome, and ab intio gene models to form a comprehensive and non-redundant reference gene set.

Gene function annotation of protein-coding genes was performed by BLASTP (with an *e*-value threshold of 1e−5 against SwissProt, Apweiler et al. 2004), GO (Gene Ontology Consortium 2017), KEGG (Kanehisa et al. 2014), PFAM (Finn et al. 2016), and InterProScan (Jones et al. 2014) databases, respectively.

We tested the quality of the final *V. cardui* annotation using an ortholog hit ratio analysis (OHR) modified from O’Neil, et al. (2010), which quantified the number and similarity of homologous proteins between our *V. cardui* annotation and a high-quality *B. mori* annotation (NCBI *B.mori* annotation release 102). We identified complete transcripts in the *V. cardui* annotation with *gffread* of the Cufflinks (Trapnell et al. 2010), collapsed both the *B. mori* and *V. cardui* proteins to nonredundant representative sequences with CD-HIT (Fu et al. 2012), and searched the collapsed *B. mori* proteins against a BLASTP (Camacho et al. 2009) database of the *V. cardui* annotation. For each *B. mori* protein, the OHR was calculated as the proportion of the *B. mori* protein covered by the longest orthologous hit. For each of these hits, we also analyzed the amino acid similarity (% identity) reported in the BLASTP output. We further compared the *V. cardui* OHR analysis results with that from another published butterfly *D. plexippus* (Danaus_plexippus.Dpv3.48.gff3.gz, updated July 11, 2020).

### Phylogenetic and Molecular Clock Analysis

To confirm the evolutionary position of *V. cardui*, OrthoFinder v1.0.6 (Li et al. 2003) was used to cluster gene families. Protein data sets from *V. cardui* and 12 related species were used for phylogenetic tree construction, including *M. cinxia*, *H. melpomene*, *B. anynana*, *D. plexippus*, *C. cecrops*, *P. sennae*, *Lerema accius*, *P. xuthus*, *B. mori*, *Plutella xylostella*, *D. melanogaster*, and *Anopheles gambiae*. All butterfly data were downloaded from LepBase (updated January 1, 2019). All-to-all BLASTP was carried out with an *e*-value threshold of 1e−5. Single-copy orthologs were subsequently aligned by MUSCLE v3.8.31 (Edgar 2004a, b). Guided by the protein multisequence alignment, the alignment of coding sequences (CDSs) for these single-copy genes were concatenated for the final data set. jModelTest v2.1.7 (Posada 2008) was used to select the best-fit model for this data set. The clade with *D. melanogaster* and *A.gambiae* was set as outgroup. RAxML v8.2.12 (Stamatakis 2015) was used to construct the phylogenetic relationships with the GTR + G + I model. MCMCtree program in PAML v4.7a (Yang 2007) was used to estimate the divergence time with the options “correlated molecular clock” and “JC69” model. Divergence time was calculated according to the fossil records, one for the split of Diptera and Lepidoptera with 290–417 Myr (Douzery, et al. 2004) and the other for the common ancestor of *D. melanogaster* and *A. gambiae* (238.5–295.4 Myr) (Benton and Donoghue 2007*)*.

### Transcriptome Analyses

The cleaned paired-end reads were aligned to the reference genome using Tophat (Trapnell et al. 2009), and reads uniquely matched to the genome were counted by htseq-count v0.13.5 (Anders et al. 2015). Global gene expression for transcripts was quantified by fragments per kilobase of transcript per million mapped reads (FPKM) using cuffquant v2.2.1 and subsequently normalized by cuffnorm v2.2.1. The principal component analysis and heatmap was performed using the PtR package of the Trinity pipeline. The average normalized FPKM value represented the corresponding quantitative gene expression level at each sample. Differential gene expression between developmental stages was measured using edgeR (Robinson et al. 2010) with biological replicates and a cutoff FDR of 0.001. 

## Supplementary Material

Supplementary data are available at *Genome Biology and Evolution* online.

## Supplementary Material

evab145_Supplementary_Data
