# Supplementary material for: High-Quality Genome Assembly and Comprehensive Transcriptome of the Painted Lady Butterfly Vanessa cardui
Source: Genome Biol Evol. 2021 Jun 28;13(7):evab145. doi: 10.1093/gbe/evab145 (PMC8290113; doi:10.1093/gbe/evab145)
Supplement: evab145_Supplementary_Data [file evab145_supplementary_data.zip › Supplementary File 1 R1.docx]

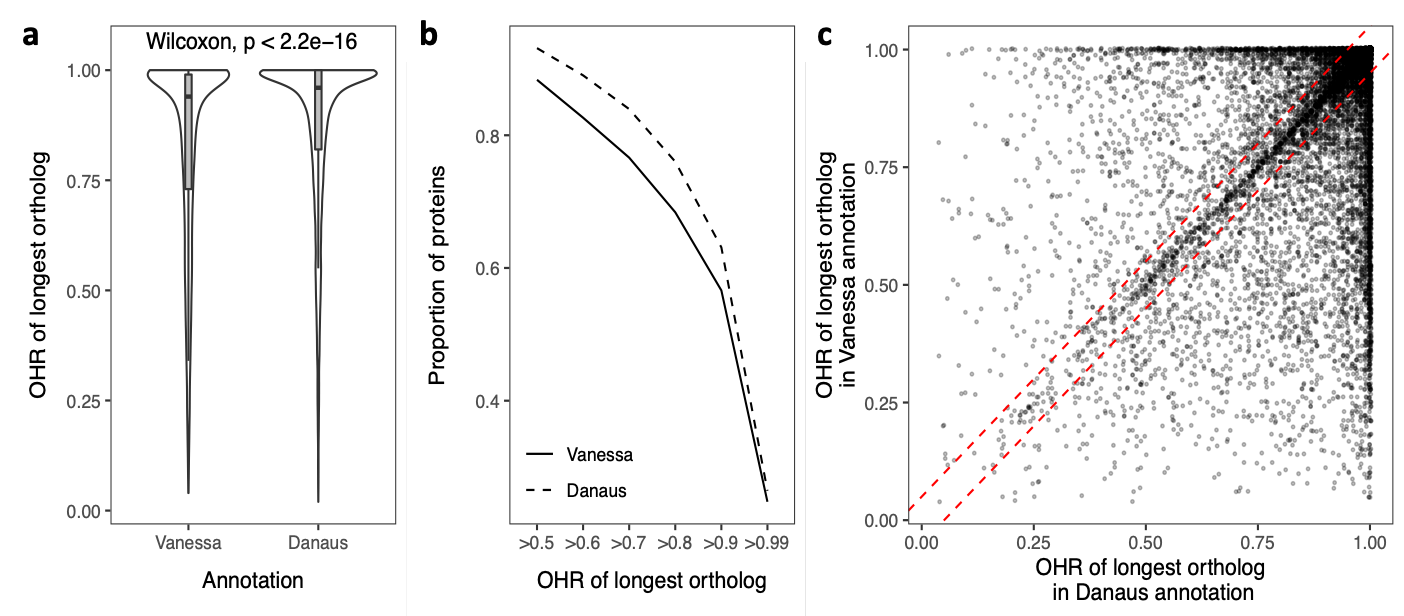


**Supplementary Figure 1.** **Genome annotation assessment.** The *V. cardui* and *D. plexippus* annotations were highly orthologous to the *B. mori* protein set, although (**a**) *V. cardui* alignments had lower ortholog hit rations (OHRs) (median = 0.94) than *D. plexippus* alignments (median = 0.96) overall, and (**b**) the cumulative proportion of *B. mori* proteins with alignments above a given OHR threshold was consistently lower for the *V. cardui* annotation (solid) than for the *Danaus* annotation (dashed). (**c**) For *B. mori* proteins with orthologs in both annotations (n = 12804), at least half of the longest orthologs were no more than 5% different between the two annotations (n = 6951). Among proteins where the OHR was more than 5% different, hits had higher identity in the Danaus annotation than in the *V. cardui* annotation (points below lower red dashed line. Violin plots in (**a**) show the scaled OHR density while boxplots visualize the upper and lower quartiles as whiskers, the middle two quartiles as gray boxes and medians as thick black bands. Outliers are not shown but can be inferred from the scaled density. OHR was estimated to the nearest 0.01. Overlapping points in (**c**) were jittered by 0.003 to reveal overlap.


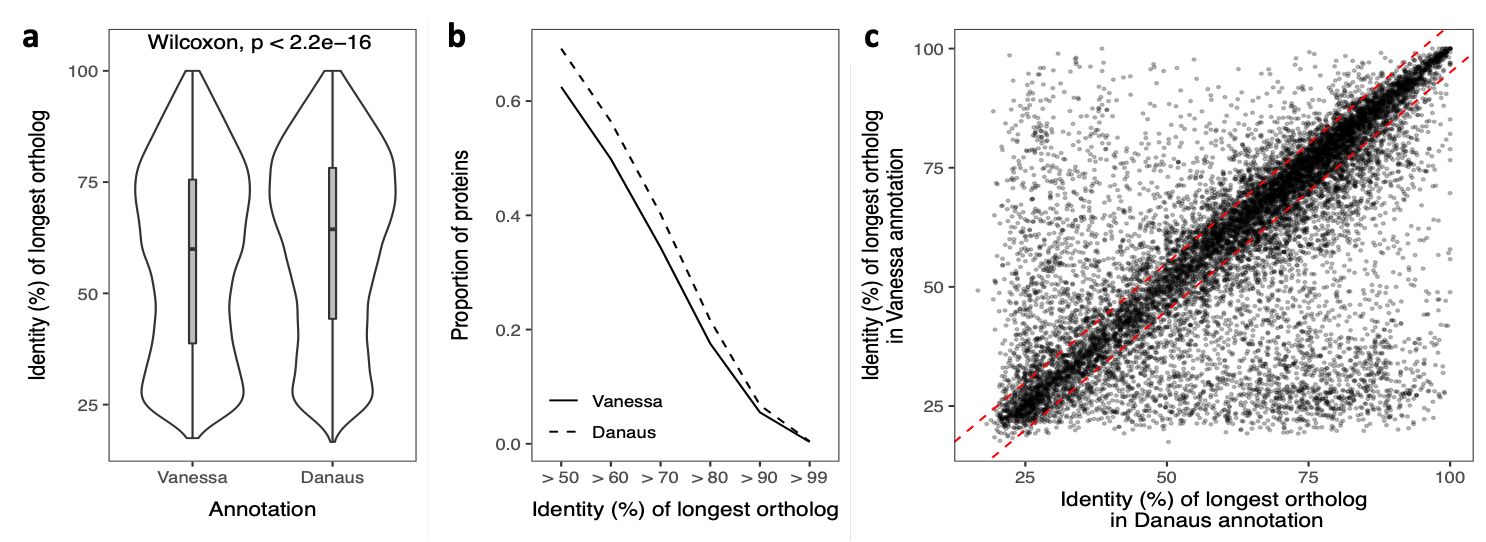


**Supplementary Figure 2.** Despite the high OHRs of the *V. cardui* and *D. plexippus* alignments, sequences were fairly divergent between the annotations and the *B. mori* protein set. (**a**) The longest *V. cardui* alignments shared lower amino acid identity (median = 59.9%) than *D. plexippus* alignments (median = 64.4%) overall, and (**b**) the cumulative number of *B. mori* proteins with alignments above a given identity threshold was consistently lower for the *V. cardui* annotation (solid) than for the *D. plexippus* annotation (dashed). (**c**) For *B. mori* proteins with orthologs in both annotations (n = 12804), identity was no more than 5% different between the two annotations (between dashed lines, n = 6726). Violin plots in (**a**) show the scaled OHR density while boxplots visualize the upper and lower quartiles as whiskers, the middle two quartiles as gray boxes and medians as thick black bands.

**
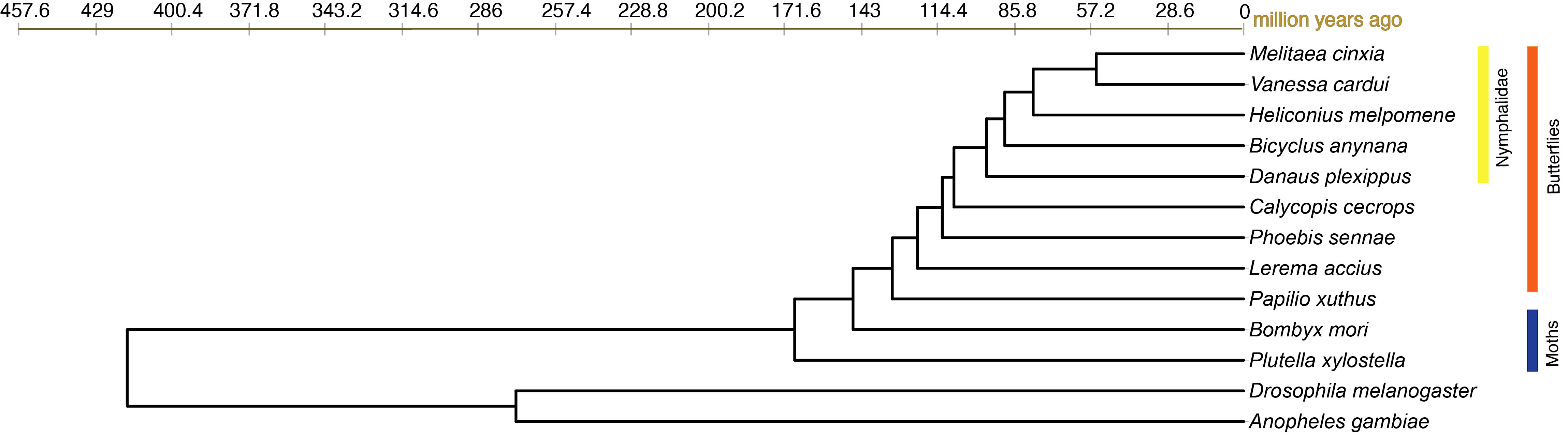
**

**Supplementary Figure 3.** Phylogenetic relationships and divergence time estimates between *V. cardui* and related species based on sequences from 326 orthologous protein coding genes.


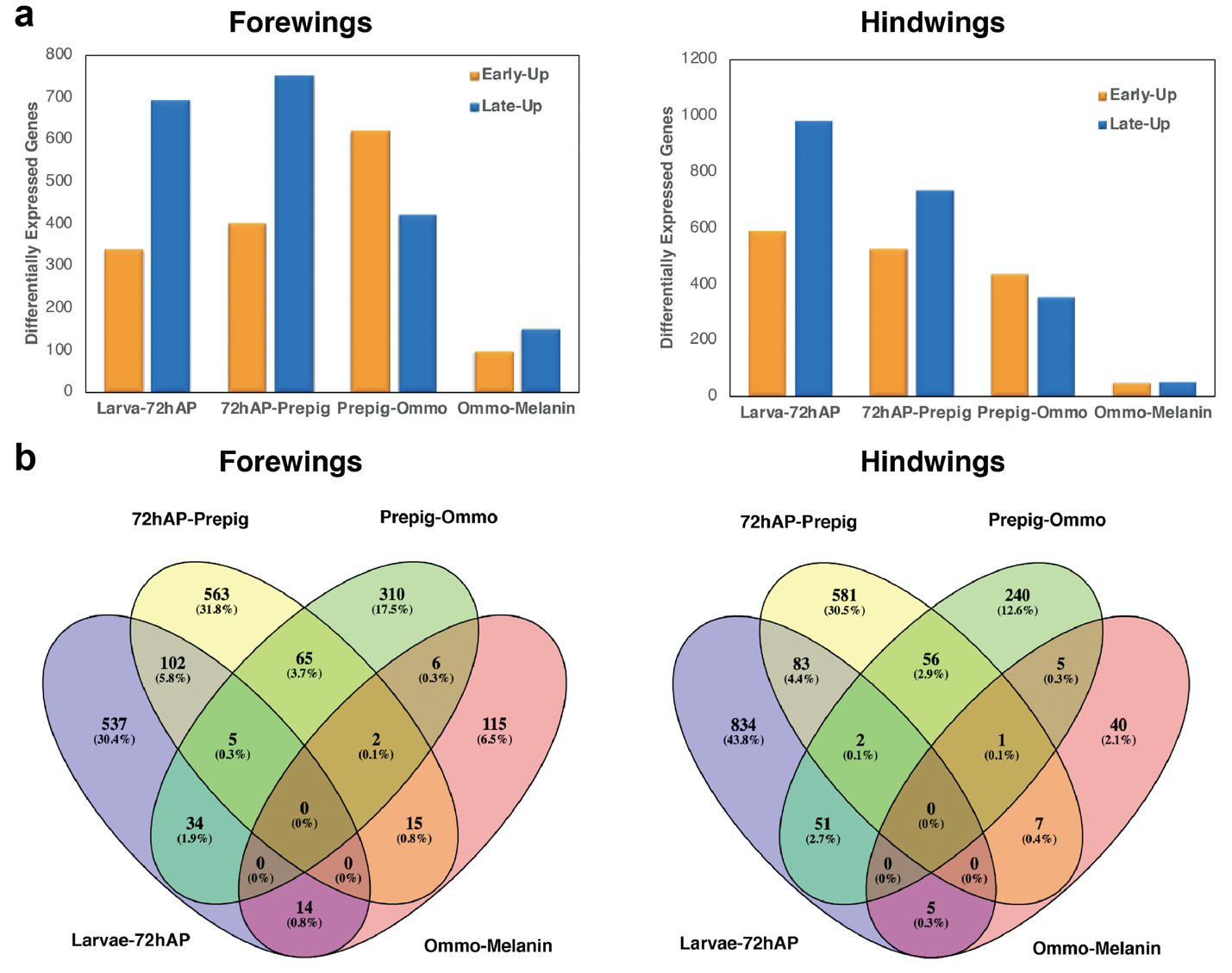
**Supplementary Figure 4.** **Summary of differential gene expression data across developmental stages.** (**a**) The number of the differentially expressed genes across stages (last instar larvae, 72h after pupation, pre-pigmentation, ommochrome stage, melanin stage). (**b**) Venn diagram of the differentially expressed genes in pairwise comparisons of consecutive stages in *V. cardui* forewings and hindwings.

**Supplementary Table 1** Summary of RNA-seq libraries and sequence data.

|  | Sample ID | Application | Sequenced reads (Mb) | Mapped reads (Mb) | Mapping ratio | Study |
| --- | --- | --- | --- | --- | --- | --- |
| Body development | Embryo 2-12hr | Annotation | 36.97 | 34.83 | 94.20% | This study |
|  | Embryo 12-52hr |  | 38.47 | 36.26 | 94.30% | This study |
|  | Larva Body |  | 36.04 | 33.15 | 92.00% | This study |
|  | Early Pupal Head |  | 46.21 | 23.64 | 93.80% | This study |
|  | Early Pupal Thorax |  | 39.24 | 34.26 | 87.30% | This study |
|  | Early Pupal Abdomen |  | 49.24 | 47.41 | 91.40% | This study |
|  | Late Pupal Head |  | 44.87 | 33.28 | 74.20% | This study |
|  | Late Pupal Thorax |  | 39.95 | 32.44 | 81.20% | This study |
|  | Late Pupal Abdomen |  | 41.50 | 35.70 | 86.00% | This study |
| Forewing development | Larva FW1 | Gene expression & annotation | 38.36 | 36.14 | 94.10% | This study |
|  | Larva FW2 |  | 40.63 | 38.22 | 94.10% | This study |
|  | 72h AP FW1 |  | 51.50 | 48.04 | 93.30% | This study |
|  | 72h AP FW2 |  | 34.19 | 31.38 | 91.80% | This study |
|  | Pre-pigment FW1 |  | 33.58 | 30.59 | 91.10% | This study |
|  | Pre-pigment FW2 |  | 32.53 | 29.89 | 91.90% | SRX1605768 |
|  | Ommochrome FW1 |  | 31.59 | 29.64 | 93.40% | This study |
|  | Ommochrome FW2 |  | 24.12 | 21.07 | 87.40% | This study |
|  | Melanin FW1 |  | 37.62 | 32.87 | 87.40% | This study |
|  | Melanin FW2 |  | 27.72 | 26.17 | 94.40% | This study |
| Hindwing development | Larva HW1 | Gene expression & annotation | 29.40 | 23.44 | 79.90% | GSM2067574 |
|  | Larva HW2 |  | 29.31 | 22.92 | 78.20% | GSM2067574 |
|  | 72h AP HW1 |  | 38.45 | 29.76 | 77.40% | GSM2067575 |
|  | 72h AP HW2 |  | 31.82 | 24.19 | 76.00% | GSM2067576 |
|  | Pre-pigment HW1 |  | 40.96 | 30.22 | 73.80% | GSM2067577 |
|  | Pre-pigment HW2 |  | 35.39 | 25.98 | 73.40% | GSM2067578 |
|  | Ommochrome HW1 |  | 46.56 | 32.75 | 70.30% | GSM2067579 |
|  | Ommochrome HW2 |  | 53.02 | 39.21 | 64.80% | GSM2067510 |
|  | Melanin HW1 |  | 6.92 | 5.01 | 72.50% | GSM2067511 |
|  | Melanin HW2 |  | 36.97 | 28.06 | 75.90% | GSM2067512 |

**Supplementary Table 2** Comparison of butterfly genome assemblies and annotation metrics.

| **Family** | **Species** | **Genome size (kb)** | **Scaffold**  **/Contig N50 (kp)** | **BUSCO**  **(%)** | **GC content**  **(%)** | **Repeat (%)** | **Number of proteins (k)** | **Note** |
| --- | --- | --- | --- | --- | --- | --- | --- | --- |
| Nymphalidae | *Vanessa cardui* | 425 | 10,297 | 97.3 | 33.37 | 34.1 | 14.4 |  |
|  | *Danaus plexippus* | 249 | 716 | 98.0 | 31.6 | 16.3 | 15.1 | v3 |
|  | *Heliconius melpomene* | 275 | 2,103 | 95.6 | 32.8 | 24.9 | 12.8 | hmel2 |
|  | *Melitaea cinxia* | 390 | 119 | 83.0 | 32.6 | 27.5 | 16.7 |  |
|  | *Bicyclus anynana* | 475 | 638 | 97.6 | 36.5 | 25.8 | 22.6 |  |
| Papilionidae | *Papilio bianor* | 421 | 13,120 | 96.3 | 36.6 | 55.3 | 15.4 |  |
|  | *Papilio xuthus* | 244 | 6,199 | 97.6 | 33.8 | 22.4 | 13.1 |  |
|  | *Papilio machaon* | 281 | 1,150 | 95.5 | 32.3 | 22.3 | 15.5 |  |
|  | *Papilio polytes* | 227 | 3,672 | 91.8 | 34.0 | 23.8 | 12.2 |  |
|  | *Papilio memnon* | 233 | 5,457 | 96.6 | 32.8 | 22.5 | 12.4 |  |
|  | *Papilio glaucus* | 375 | 231 | 95.5 | 35.4 | 22.0 | 15.7 |  |
| Hesperiidae | *Achalarus lyciades* | 567 | 558 | 97.3 | 35.3 | 25.0 | 15.9 |  |
|  | *Lerema accius* | 298 | 525 | 95.1 | 34.4 | 15.5 | 17.4 |  |
|  | *Megathymus ursus violae* | 429 | 4,153 | 98.3 | 34.7 | 25.8 | 14.1 |  |
| Pieridae | *Pieris rapae* | 246 | 617 | 98.0 | 32.7 | 22.7 | 13.2 |  |
|  | *Phoebis sennae* | 406 | 257 | 97.7 | 39.0 | 17.2 | 16.5 |  |
| Riodinidae | *Calephelis nemesis* | 809 | 206 | 95.6 | 34.9 | 34.8 | 15.4 |  |
|  | *Calephelis virginiensis* | 855 | 175 | 93.9 | 35.0 | 38.8 | 15.6 |  |
| Lycaenidae | *Calycopis cecrops* | 729 | 233 | 95.5 | 37.1 | 34.0 | 16.5 |  |
